# A comparative study of Pentacam indices in various types and severities of refractive error in candidates for photorefractive keratectomy (PRK) surgery

**DOI:** 10.25122/jml-2021-0027

**Published:** 2022-06

**Authors:** Ghazal Maraghechi, Habib Ojaghi, Firouz Amani, Amin Najafi

**Affiliations:** 1 Department of Medical Education, School of Medicine, Ardabil Azad University, Ardabil, Iran; 2 Department of Surgery, School of Medicine and Allied Medical Sciences, Ardabil University of Medical Sciences, Ardabil, Iran; 3 Department of Community Medicine, School of Medicine and Allied Medical Sciences, Ardabil University of Medical Sciences, Ardabil, Iran

**Keywords:** refractive error, hyperopia, astigmatism, myopia, PRK, Pentacam indices

## Abstract

This study aimed to specify Pentacam indices in patients who suffered from different types of refractive error and underwent photorefractive keratectomy (PRK) surgery. It is a descriptive cross-sectional study carried out on 1125 patients (2215 eye samples) who underwent PRK surgery in the Noor Surgical Center of Ardabil, Iran, over a 5 year period (2014–2018). A particular checklist was provided to patients, which consisted of demographic data, pachymetry test, keratometry, refractive error, corneal-thickness indices, and corneal surface area indices. The data were analysed using the statistical analysis package of IBM^®^ V25. The mean age of the participants in this study was 28.48±6.82 years, and the ratio of women to men was 66.4%. It was observed that the differences between angle, volume, the depth of the anterior chamber, IVA, and ISV were significant (P=0.00) when compared to each other in all types of refractive errors. High myopes had significantly higher K_max_ front than low myopes (P=0.00). In astigmatism patients, the K_max_ in front of the cornea in extreme type was significantly higher than in moderate (P=0.00) and high (P=0.01) types. High myopes had significantly lower R_min_ than mild myopes (P=0.02), and extreme astigmatism had significantly lower R_min_ than high (P=0.014) and moderate types (P=0.013). The data from this study revealed that in patients undergoing PRK surgery, some Pentacam indices could be related to some types of refractive error, and in some of these indices, there are statistically significant differences between different severities of refractive errors. Therefore, their preoperative evaluation is very important.

## INTRODUCTION

Given the prevalence of refractive error, photorefractive keratectomy (PRK) surgery has been the most common non-emergency eye treatment option in the last two decades. The procedure of these types of surgeries consists of correction of astigmatism, hyperopia, and myopia and the decrement of spectacles dependence and reduction of dependence on contact lenses [1–4].

However, ophthalmologists face numerous challenges in identifying patients at risk for postoperative complications. These complications mainly include ectasis, undercorrection, and overcorrection [5, 6]. Therefore, the errors induced from corneal indices measurement could be minimised using diagnostic instruments such as a Galilei analyzer, ultrasonography, Orbscan corneal topography, and Pentacam system. The Pentacam system is among the most prevalent instruments used in this regard [7]. One of the most crucial steps in achieving a suitable therapeutic strategy to prevent PRK complications is to measure corneal parameters accurately.

Consequently, in refractive surgery, the initial basis for diagnostic tests is determining the topographic design for different types of refractive errors. In addition, in every country, there is a need for a comprehensive database on the characteristics of the components of the visual system because of the effect of geographic and racial factors. The present study aimed to examine and compare various Pentacam indices in patients with different types and severities of refraction error who underwent the PRK surgical procedure at Noor ophthalmology clinic in Ardabil, Iran, for 5 years from the beginning of 2014 until the end of 2018.

## MATERIAL AND METHODS

The present study is a descriptive cross-sectional study of patients who underwent PRK surgery between 2014 and 2018 (5 years) at Noor Surgery Center in Ardabil, Iran (a private ophthalmology practice setup). The sampling was done using the census method, based on which 1125 patients were selected, and finally, 2215 eyes were examined. The data of patients consisting of demographics (age and gender), keratoconus (KCN) classification, pachymetric, keratometric, and refractive data were obtained based on the records of patients and then included in a detailed checklist. A skilled surgeon (Ojaghi H) examined the patients before the operation and then performed the operation. A new Canon RK-F2 Full Auto Ref-Keratometer made in Tokyo, Japan was used to perform a refraction test 30 minutes after instilling two drops of cyclopentolate 5 minutes apart. The data obtained from examining the eye using a Pentacam scanner (Oculus Instruments, Wetzlar, Germany) includes corneal topography, corneal pachymetry, and assessment of the anterior chamber angle (ACA). Anterior chamber depth also included corneal thickness.

The following rules were used to specify refractive astigmatism and anterior and posterior corneal astigmatism based on the steep axis of corneal astigmatism.

Based on previous studies, with-the-rule (WTR) astigmatism is between 0°–30° or 150°–180°, against-the-rule (ATR) astigmatism is between 60°–120°, and oblique astigmatism (OA) is between 31°–59° or/and 121°–149° [8, 9].

The amount of corneal astigmatism was determined using the following formula [10]:

Corneal astigmatism = maximum keratometry (K_2_) - minimum keratometry (K_1_)

Refraction error was divided based on intensity levels and myopia was categorized into four levels as follows:

1. Mild myopia: When the amount of lens power (sphere) is less than 3.00 diopters;
2. Moderate myopia: When the amount of lens power (sphere) is between 3.00 to 6.00 diopters;
3. High myopia: When the amount of lens power (sphere) is between 6.25 to 9.00 diopters;
4. Extreme myopia: When the amount of lens power (sphere) is more than 9.00 diopters [11, 12].

Hyperopia was categorized into three levels as follows [13]:

1. Low hyperopia: When the amount of lens power (sphere) is up to + 2 diopters;
2. Moderate hyperopia: When the amount of lens power (sphere) is between 2.25 and 4.74 diopters;
3. High hyperopia: When the amount of lens power (sphere) is more than 5.00.

Based on its severity, astigmatism was categorized as follows:

1. Mild astigmatism: When its degree is less than 1 diopter;
2. Moderate astigmatism: When its degree is between 1 to 2 diopter;
3. High astigmatism: When its degree is between 2.25 to 4 diopters;
4. Extreme astigmatism: When its degree is more than 4 diopters [12].

Inclusion criteria included stable refraction with a change of less than 0.5 diopters (D) of myopia, hyperopia, and astigmatism in the last 6 months and age ≥18 years at the time of surgery.

Patients with a history of HSV (herpes simplex virus) keratitis, blepharitis, immunosuppressive diseases, uncontrolled diabetes, corneal scar, severe dry eye, herpes keratitis, uveitis, cataract, and people with thin corneas (thickness less than 480 µm or expected postoperative residual stromal thickness (RST) less than 300 µm) were excluded from the study.

### Statistical analysis

The data of the present study were analysed using SPSS software version 25.0. In the majority of tables and graphs, the index of dispersion (variance, standard deviation, range) and the central tendency (mean, median, mode) descriptive statistics were used. In addition, student's t-test, Pearson correlation coefficient, and one-way analysis of variance (ANOVA) were used to perform analytical statistics. A p-value less than 0.05 was considered statistically significant (typically P≤0.05).

## RESULTS

Our study was carried out on 1125 patients (2215 eye samples) who underwent PRK surgery with a female to male ratio of 66.4% and mean age of 28.48±6.82 years (ranging from 18 to 52 years, median of 27 years, and mode of 24 years) among which 1111 participants were right eyes (50.2%). The score of the sphere ranged from -10.5 to +8.5 diopters with a mean of -3.39±2.55D, and refractive astigmatism ranged from 0–6 diopters with a mean of -1.03±1.12D. The spherical equivalent among samples ranged from -10.75 and +7.50 diopters with a mean of -3.91±2.50D.

**Figure 1:**
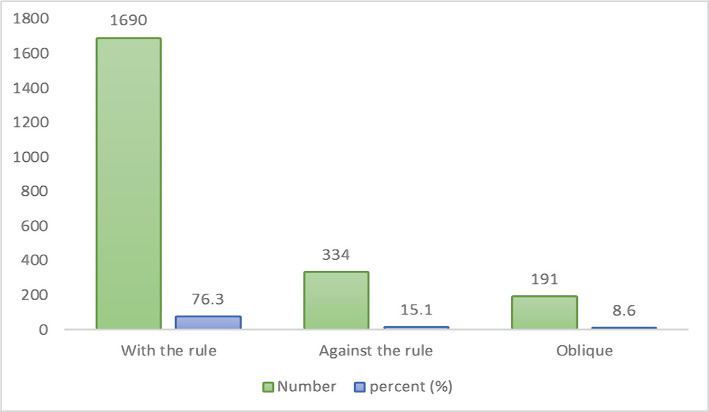
Frequency and percentage of various types of astigmatism.

Figure 1 represents the frequency and percentage of refractive astigmatism in accordance with the steepest meridian in the studied samples. In our study, the most prevalent type of refractive astigmatism was with-the-rule (WTR) astigmatism (76.3%).

**Figure 2:**
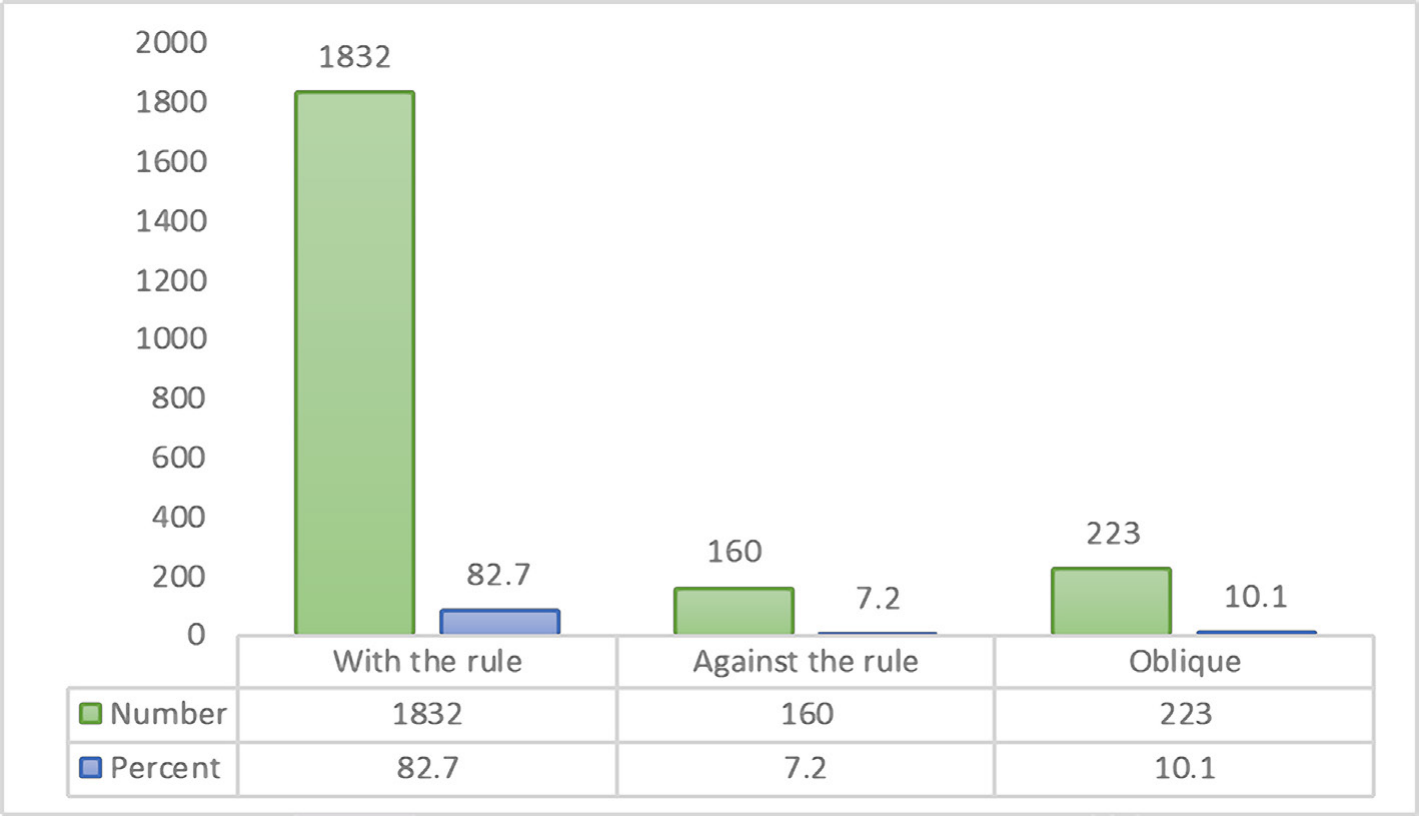
Different types of anterior corneal astigmatism (frequency and percentage).

In 82.7% of participants (1832 individuals), the anterior corneal astigmatism was WTR followed by ATR (7.2%; 160 eyes) and oblique (10.1%; 223 eyes) (Figure 2).

**Figure 3:**
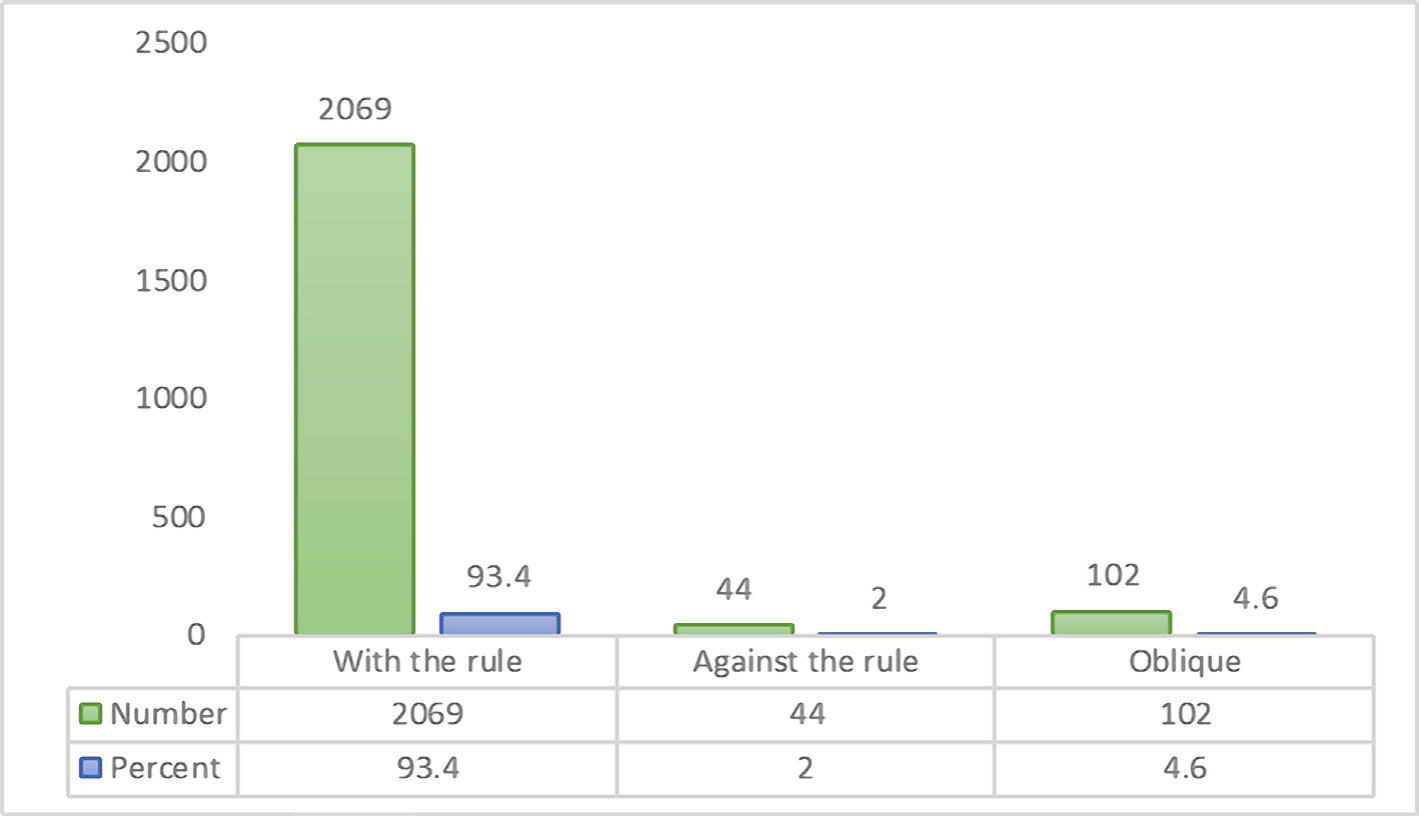
Different types of posterior corneal astigmatism (frequency and percentage).

As could be seen from Figure 3, in 93.4% of cases (2069 eyes), the posterior corneal astigmatism was type WTR, followed by ATR (2%; 44 eyes) and oblique (4.6%; 102 eyes).

Tables 1 and 2 represent the anterior chamber indices and keratometric indices, including anterior and posterior corneal surface areas (As/Ps) (k_1_, k_2_, and k_mean_). The mean posterior corneal astigmatism is -0.34±0.18D, and mean anterior corneal astigmatism is -1.11±1.14D.

**Table 1:** Keratometric indices.

	Keratometric indices	Mean±SD	Range
**Anterior cornea**	K_1_	43.09±1.52	37.8–48.5
	K_2_	44.39±1.52	39.7–50.1
	K_mean_	43.73±1.45	39.2–49
	Astigmatism (K_2_-K_1_)	-1.116±1.14	-6.1–4.9
	K_max_	44.84±1.58	40.3–54.4
**Posterior cornea**	K_1_	-6.09±0.24	(-7.2)–(-5.4)
	K_2_	-6.44±0.27	(-7.5)–(-5.6)
	K_mean_	-6.26±0.24	(-7.3)–(-5.6)
	Astigmatism (K_2_-K_1_)	-0.34±0.18	-1.1–0.4

**Table 2:** The data achieved from the corneal pachymetry technique and anterior chamber indices.

Indices	Range	Mean±SD
**Apex pachymetry (µm)**	434–687	534.26±32.04
**Thinnest location pachymetry (µm)**	429–680	531.27±32.48
**Cornea volume (mm^3^)**	49.5–76.2	59.64±3.61
**Chamber volume (mm^3^)**	94–336	201.62±35.13
**Anterior chamber depth (mm)**	2.67–6.68	3.78±0.30
**Anterior chamber angle (degree)**	15.1–79.5	37.94±6.21

SD – Standard deviation.

The data from Pearson's correlation coefficient test showed a strong direct significant association (P=0.00, r=99%) between the thinnest location and the pachymetry of the apex. Myopia was observed in 85.73% of eye cases (1899 eyes), astigmatism in 10.57% (234 eyes), and hyperopia in 3.7% of them (82 eyes).

In 56.5% of cases, myopia was moderate, followed by 27.96% mild myopia, 14.69% of cases with high myopia, and 0.85% with extreme myopia. Also, 6%,47.6%, and 46.4% of hyperopic eyes had low, moderate, and high hyperopia, respectively. In 20.8% of cases, astigmatism was extreme, in 54.7% high, 24.7% moderate, and 0% mild.

Based on the location of the focal lines in relation to the retina, the percentages of the astigmatism types were as follows: 63.24% were compound (148 cases), 25.64% simple (60 cases), and 11.12% were mixed (26 cases).

Based on the data presented in Table 3, the association of refractive errors of myopia, hyperopia, and astigmatism with R_min_, IHA, IVA, ISV, AC depth, angle, cornea volume, K_max_ front, chamber volume, and mean thinnest location indices is significant. However, the association of refractive errors with the thinnest location was weak. Moreover, the association of refractive errors with indices of pachymetry, including KI, CKI, IHD, Prog_min_, Prog_avg_, and Prog_max_, was not significant.

**Table 3:** The association of various types of refractive error with Pentacam indices.

Pentacam indices	Refractive errors M±SD			P-value
	Myopia n=1899 (85.73%)	Astigmatism n=234 (10.57%)	Hyperopia n=82 (3.70%)	
**AC Depth**	3.80±0.28	3.72±0.39	3.38±0.31	0.00
**Chamber volume**	204.52±33.78	191.75±34.25	156.95±35.07	0.00
**Angle**	38.27±6.13	36.76±6.36	33.62±5.79	0.00
**ISV**	15.79±4.95	26.75±7.82	19.91±8.29	0.00
**IVA**	0.10±0.06	0.12±0.05	0.14±0.09	0.00
**IHA**	3.041±2.386	4.109±3.327	3.850±2.986	0.00
**IHD**	0.005±0.009	0.007±0.003	0.007±0.004	0.08
**KI**	1.020±0.031	1.019±0.018	1.015±0.019	0.247
**CKI**	1.007±0.006	1.007±0.005	1.006±0.005	0.20
**Cornea volume**	59.72±3.59	59.11±3.80	59.26±3.21	0.006
**Pachy apex**	534.39±31.99	530.94±33.56	540.52±27.69	0.059
**Thinnest location**	531.42±31.87	527.04±34.22	536.23±27.51	0.048
**Rmin**	7.539±0.286	7.439±0.268	7.665±0.272	0.00
**K_max_ front**	44.80±1.54	45.42±1.63	44.19±1.94	0.00
**Prog_min_**	0.685±0.128	0.688±0.130	0.672±0.101	0.62
**Prog_avg_**	0.953±0.125	0.960±0.129	0.929±0.117	0.156
**Prog_max_**	1.180±0.177	1.191±0.168	1.157±0.150	0.329

M – Mean; SD – standard deviation; ACD – anterior chamber depth; R_min_ – minimum radius of curvature; Prog_min_ – pachymetric progression index minimum; Prog_avg_ – pachymetric progression index average; Prog_max_ – pachymetric progression index maximum; KI – keratoconus index; CKI – central keratoconus index; IHD – index of height decentration; IHA – index of height asymmetry; IVA – index of vertical asymmetry; ISV – index of surface variance.

Table 4 shows the relationship between Pentacam indices in various types of refractive errors. For instance, the differences in angle, volume, IVA, ISV, and anterior chamber depth were significant (p=0.00) compared to each other in all types of refractive errors (except for the comparison of IVA between hyperopia and astigmatism with p=0.057)

**Table 4:** Paired intergroup comparison of Pentacam indices.

Indices	Myop n=1899	Hyperop n=82	P-value	Hyperop n=82		Ast n=234	P-value	Myop n=1899		Ast n=234	P-value
	Mean±SD			Mean±SD				Mean±SD			
**Thinnest location (µm)**	531.42±31.87	536.23±27.51	0.378	536.23±27.51	527.04±34.22		0.065	531.42±31.87	527.04±34.22		0.118
**Kmax front (D)**	44.80±1.54	44.19±1.94	0.002	44.19±1.94	45.42±1.63		0.00	44.80±1.54	45.42±1.63		0.00
**Cornea volume (mm^3^)**	59.72±3.59	59.26±3.21	0.491	59.26±3.21	59.11±3.80		0.950	59.72±3.59	59.11±3.80		0.042
**AC depth (mm)**	3.804±0.281	3.382±0.311	0.00	3.382±0.311	3.726±0.396		0.00	3.804±0.281	3.726±0.396		0.00
**Chamber volume (mm^3^)**	204.52±33.78	156.95±35.07	0.00	156.95±35.07	191.75±34.25		0.00	204.52±33.78	191.75±34.25		0.00
**Angle (degree)**	38.273±6.130	33.620±5.793	0.00	33.620±5.793	36.766±6.365		0.00	38.273±6.130	36.766±6.365		0.00
**ISV**	15.798±4.953	19.914±8.297	0.00	19.914±8.297	26.752±7.821		0.0	15.798±4.953	26.752±7.821		0.00
**IVA (mm)**	0.104±0.061	0.140±0.093	0.00	0.140±0.093	0.122±0.050		0.057	0.104±0.061	0.122±0.050		0.00
**IHA (µm)**	3.041±2.386	3.850±2.986	0.013	3.850±2.986	4.109±3.327		0.703	3.041±2.386	4.109±3.327		0.00
**Rmin (mm)**	7.539±0.286	7.665±0.272	0.00	7.665±0.272	7.439±0.268		0.00	7.539±0.286	7.439±0.268		0.00

SD – Standard deviation; Myop – myopia; Hyperop –hyperopia; Ast – astigmatism; AC depth – anterior chamber depth; R_min_ – minimum radius of curvature; IHA – index of height asymmetry; IVA – index of vertical asymmetry; ISV – index of surface variance.

Table 5 shows the relationship between Pentacam indices and different severities of refractive errors. The difference in mean±SD of the K_max_ front between severities in both myopia (p=0.003) and astigmatism (p=0.00) is significant. The intra-group analysis demonstrated that, in myopes, only high myopes had significantly higher K_max_ front than low myopes (p=0.00). In astigmatism, all three groups had significant differences, and maximum keratometry (K_max_) was significantly higher in extreme type in comparison with moderate (p=0.00) and high (p=0.01) type and in high astigmatism than moderate types (p=0.02). The AC depth in myopes was significantly higher than that of astigmatism and hyperopia, and in astigmatism it was significantly higher than hyperopes (all three p=0.00). However, in intra-group variance analysis, the differences between different refractive error severities in AC depth were not significant.

**Table 5:** Comparing Pentacam indices based on refractive severity.

	Myopia				P-value	Hyperopia			P-value	Astigmatism			P-value
	Mild n=531	Moderate n=1073	High n=279	Extreme n=16		Low n=5	Moderate n=39	High n=38		Moderate n=58	High n=128	Extreme n=48	
**K_max_ front (D)**	44.63±1.49	44.81±1.54	45.05±1.56	44.81±1.50	0.003	43.70±2.05	44.09±2.21	44.36±1.64	0.70	44.75±1.53	45.44±1.59	46.20±1.52	0.00
**Cornea volume (mm^3^)**	59.57±3.54	59.76±3.73	59.76±3.16	61.27±2.87	0.25	59.64±2.82	58.47±2.85	58.99±3.63	0.781	58.72±3.60	59.36±3.89	58.94±3.83	0.543
**Chamber volume (mm^3^)**	201.12±33.58	206.32±34.58	204.88±31.80	190.81±25.10	0.011	149.40±27.66	158.33±33.11	156.52±38.40	0.865	198.27±35.33	192.10±33.38	192.66±35.45	0.510
**AC depth (mm)**	3.785±0.273	3.816±0.284	3.802±0.283	3.650±0.223	0.028	3.342±0.244	3.413±0.282	3.356±0.348	0.699	3.832±0.526	3.704±0.357	3.655±0.280	0.049
**Angle (degree)**	38.490±6.091	38.406±6.124	37.457±6.233	36.413±5.034	0.055	35.660±3.017	33.577±5.922	33.395±5.987	0.717	38.972±5.484	36.150±6.663	35.744±6.023	0.009
**ISV**	14.871±4.562	15.687±4.890	17.863±5.398	17.937±2.112	0.00	19.600±4.979	17.871±6.817	22.052±9.549	0.085	19.000±4.120	26.835±5.112	35.895±7.247	0.00
**IVA**	0.104±0.043	0.102±0.058	0.111±0.093	0.103±0.038	0.142	0.168±0.031	0.116±0.046	0.162±0.125	0.080	0.107±0.040	0.121±0.052	0.142±0.048	0.001
**IHA**	2.917±2.343	3.028±2.324	3.305±2.677	3.443±2.458	0.149	2.600±2.867	3.430±2.609	4.444±3.298	0.209	3.256±2.668	4.211±3.468	4.866±3.497	0.04
**R_min_ (mm)**	7.563±0.340	7.538±0.261	7.500±0.262	7.541±0.255	0.034	7.744±0.377	7.701±0.256	7.617±0.275	0.329	7.522±0.261	7.436±0.261	7.313±0.242	0.00

K_max_ front – maximum keratometry of anterior cornea; D – Diopter; R_min_ – minimum Radius of curvature; AC depth – anterior chamber depth; IHA – index of height asymmetry; IVA – index of vertical asymmetry; ISV – index of surface variance.

In the anterior chamber volume analysis, only moderate myopia had a significantly higher volume in comparison with the mild myopia(p=0.019), and only in astigmatism, moderate types had higher anterior chamber angle in comparison with the high (p=0.012) and extreme (p=0.024) types.

The ISV of moderate myopes was significantly higher compared with the mild types (p=0.00), and high myopes had a significantly higher ISV in comparison with ISV of moderate and mild types (p=0.00). Besides, the ISV of high astigmatism was significantly higher compared with moderate types (p=0.00), and the ISV of extreme astigmatism was significantly higher in comparison with high and moderate types (p=0.00).

In the analysis of asymmetric variables of the cornea, only extreme astigmatism had significantly higher IVA and IHA than moderate types (p=0.001 and p=0.035, respectively).

Eventually, high myopes had significantly lower R_min_ than mild myopes (p=0.02). Also, extreme astigmatism had significantly lower R_min_ than high (p=0.014) and moderate (p=0.00) types, and high astigmatism had significantly lower R_min_ than moderate types (p=0.013). In other indices, the differences between different severities of refractive errors in terms of Pentacam indices were not significant.

## DISCUSSION

In our study, the participants ranged from 18–52 years with a mean age of 28.48±6.82, and 66.4% were female. The mean spherical equivalent of the participants' eyes was -3.91±2.50D. Moreover, the prevalence of myopia was the highest (85.73%), followed by astigmatism (10.57%) and by hyperopia (3.70%). As reported by Seyed Javad Hashemian et al. [14], 91.95% of the 2673 cases who were screened for refractive surgery were detected with myopia.

In another study, Heydari et al. [15] showed that 94.2% of 400 studied eyes had myopia as the most serious vision problem, and the least was hyperopia with a percentage of 3.3%. In their study, the mean spherical equivalent was -3.29±2.27D, which is in line with the data achieved in our study. These results could be justified by the better response of myopia to PRK compared with the other types of refractive errors. In our study, the mean posterior and anterior corneal K_1_ were -6.099±0.24D and 43.097±1.52D, respectively. Moreover, the mean posterior and anterior corneal K_2_ were -6.442±0.27D and 44.394±1.52D, respectively. In addition, the association of the K_max_ front with the refractive errors was significant, and the mean K_max_ front was 44.844±1.58D (Tables 1–5).

Based on the data from this study, it was observed that the mean K_max_ front for hyperopia and myopia was 44.19±1.94D and 44.80±1.54D, respectively. In a similar study by Hashemi et al. [16], who evaluated anterior chamber criteria regarding the refractive status of patients, 283 eye samples were divided into three groups (hyperopia, myopia, and emmetropia), and those with a myopic disorder were divided into four subgroups. Based on their study, it was observed that 85% of samples were detected with myopia. However, in contrast to the data of our study, the differences in refractive errors and K_max_ Front were not significant (p=0.1), with a mean of 44.3±2.2D for hyperopia and 45.03±1.44D for myopia. Although there was no difference between the K_max_ front in the hyperopia and myopia, the K_max_ front in the myopia was higher than the hyperopia. The lack of mentioned difference could be explained by the higher powers of myopic eyes when compared with the hyperopes, which is to some extent due to the high corneal power in myopes.

In our study, the average thickness of cornea at thinnest and apex locations were 531.279±32.48 µm and 534.261±32.04 µm, respectively. There was a substantial significant association between the mentioned variables (P=0.00). However, the association of these two variables with refractive errors was not statistically significant. In their study, Mohammadi et al. [17] revealed a significant association between refractive error and corneal thickness in hyperopes. In another study by Mahmoud et al. [18], a significant association was reported between the severity of myopia and the central corneal thickness (CCT). However, these two studies mentioned above contradict our study. Hashemi et al. [16] observed no significant association between the thinnest location and corneal thickness with refractive errors. In another study, a mean of 549.5±33.6 µm was reported for CCT, while the association of CCT with refractive errors was not significant [19].

In addition, Fam et al. [20] reported no significant association between CCT and the degree of myopia in their study. The data in our study correspond with the findings of the three studies mentioned above and some other studies in which the association of CCT and refractive errors was not significant [21–23]. The association between refractive errors and corneal thickness could be explained by geographical or racial differences in various populations.

Our study revealed that the association of anterior chamber indices and refractive errors is statistically significant, as, in the analysis of AC depth and volume, there was a significant difference between hyperopia with both myopia and astigmatism and also between astigmatism and myopia (Table 4). Intra-group analysis of cornea volume demonstrated a significant difference only between astigmatism and myopia. Our study revealed that patients in the myopia group had the highest amount of angle, chamber volume, and AC depth. Furthermore, there was a significant relationship between different severities of refractive errors and anterior chamber indices.

In their study, Razmjoo et al. [24] showed that the depth, volume, and the mean anterior chamber angle in patients undergoing PRK surgery were 207±50 mm^3^, 3.29±0.4 mm, and 39.7±9.2°, respectively. Another study by Hashemi et al. [16] demonstrated that the association of angle, anterior chamber volume, and anterior chamber depth with refractive errors was significant. Based on their data, the anterior chamber depth and volume in the myopes had the highest values of data. However, there was no significant association between refractive errors and cornea volume (p>0.05). A study consisting of 149 patients (297 eye samples) using Pentacam indices observed that 242 eye samples had the highest prevalence rate of myopia, and the association of pachymetric and anterior chamber indices with all refractive errors was significant (p<0.05). Moreover, the participants in the myopia group had the highest depth and volume of the anterior chamber and the lowest values of corneal volume [25]. Another study by Alrajhi et al. [26] revealed a weak correlation between myopia severity and cornea volume. Moreover, various studies reported similar findings [27–29]. Our data correspond with the studies mentioned earlier due to the larger size and volume of the eyes. So, the anterior chamber angle and depth in myopes were higher compared with hyperopes.

Despite the significant association of AC depth with myopia and astigmatism in our study, the differences between AC depth and various severities of myopia and astigmatism were not significant in the intra-group comparison (Table 5). However, in their study, Hashemi H et al. [30] showed a significant association between moderate and severe levels of hyperopia with this variable [30].

Moreover, the evaluation of surface zone indices and criteria aimed to detect keratoconus, including KI, ISV, IHA, IHD, IVA, CKI, R_min_, and their significance level of association with refractive errors were among other achievements of the present study. The overall mean ISV index was 17.1079, the mean IVA was 0.1075, R_min_ was 7.5339, and the mean IHA was 3.1843. Moreover, the data revealed a significant association between refractive errors and their severity levels with R_min_, IHA, ISV, and IVA indices. There was a significant correlation between ISV and R_min_ with different severities of myopia and astigmatism and between IHA and IVA with different levels of astigmatism severity. However, the association of refractive errors with CKI, KI, and IHD indices was not significant. A review of the literature revealed no similar data in this regard, despite the study by Brizl et al. [31], who showed the same data as ours. In patients without keratoconus (KC) disorder, the mean ISV in 36 eye samples was 21.83±8.03, their mean of IVA was 0.12±0.04, with a mean IHA of 5.02±4.29, and R_min_ of 7.18±0.17. In their study, the refractive errors were not compared with the indices.

In our study, the mean progressive corneal thickness indices were also evaluated, including Prog_min_ (0.685), Progavg (0.952), and Prog_max_ (1.180). The association of progressive corneal thickness indices and refractive errors was not significant. A study by Hashemi et al. [16] revealed that the relationship between refractive errors and Prog avg was not significant. This could be clarified using a normal pattern of gradual increment in corneal thickness from center to corneal periphery in all types of refractive errors, even in normal eyes.

According to the data achieved from this study, the mean anterior and corneal posterior astigmatism were -1.116±1.14D and -0.34±0.18D, respectively. WTR was the most prevalent type of astigmatism in both the anterior and posterior cornea. Besides, the association of anterior and posterior corneal types of astigmatism and type of refractive astigmatism was significant (p=0.00). There also was a significant association between posterior corneal astigmatism and anterior corneal astigmatism (p=0.00). Based on previous studies, the value of posterior corneal astigmatism (PCA) in the normal population ranges between 0.26 and 0.78 D [32, 33]. On the other hand, Nemeth et al. [34] reported that WTR was the most prevalent type of posterior and anterior astigmatism. In addition, Feizi et al. [35] revealed a significant association between PCA and ACA that is consistent with our study. Askari Zadeh et al. [36] conducted a retrospective case series study including 161 patients (161 eye samples) with Keratoconus at Farabi Hospital in Tehran. Based on their study, the mean posterior and anterior corneal astigmatism were 0.86±0.45D and 4.08±2.21D, respectively. Moreover, the prevalence of ATR astigmatism in the posterior cornea and WTR in the anterior corneal surface were significantly higher. Contrary to the data achieved from our study, some of the previous studies revealed that ATR and WTR was the most prevalent type of posterior and anterior corneal astigmatism, respectively [35–38].

The data presented in a study by Miyake et al. [38] revealed no significant association between ACA and PCA, which is in contrast with the results of our study. Moreover, in their study, the most common types of astigmatism were ATR astigmatism in the posterior cornea and WTR in the anterior cornea with 91% and 68%, respectively. The reported differences in the data achieved from various studies could be due to the differences in various types of posterior and anterior astigmatism based on different geographical areas and races or may be because of the availability of unknown cases which should be investigated with more detail in further studies. Eventually, one of the advantages of the present study was that all preoperative clinical and paraclinical examinations were performed only by one surgeon and a Pentacam device. Our study had no specific limitation.

## CONCLUSION

The data in this study revealed that, in patients who underwent PRK surgical procedure, Pentacam indices, including surface zone indices, anterior chamber indices, and keratometric indices, may depend on the severity and types of refractive errors. Furthermore, it should be noted that a more desirable outcome from refractive surgery could be obtained through a more accurate and effective examination of the anterior and posterior cornea.
